# Modifying the Ambient Light Spectrum Using LED Lamps Alters the Phenolic Profile of Hydroponically Grown Greenhouse Lettuce Plants without Affecting Their Agronomic Characteristics

**DOI:** 10.3390/plants13172466

**Published:** 2024-09-03

**Authors:** Cristian Hernández-Adasme, Herman Silva, Álvaro Peña, María Gabriela Vargas-Martínez, Carolina Salazar-Parra, Bo Sun, Víctor Escalona Contreras

**Affiliations:** 1 Centro de Estudios de Postcosecha (CEPOC), Departamento de Producción Agrícola, Facultad de Ciencias Agronómicas, Universidad de Chile, Santiago 8820808, Chile; criherna@ug.uchile.cl; 2 Laboratorio de Genómica Funcional y Bioinformática, Departamento de Producción Agrícola, Facultad de Ciencias Agronómicas, Universidad de Chile, Santiago 8820808, Chile; hesilva@uchile.cl; 3 Departamento de Agroindustria y Enología, Facultad de Ciencias Agronómicas, Universidad de Chile, Santiago 8820808, Chile; apena@uchile.cl; 4 Laboratorio de Desarrollo de Métodos Analíticos, Facultad de Estudios Superiores Cuautitlán, Universidad Nacional Autónoma de México, Cuautitlán Izcalli 54740, Mexico; gvargasm@unam.mx; 5 Instituto de Investigaciones Agropecuarias (INIA), Centro Regional La Platina, Santiago 8831314, Chile; carolina.salazar@inia.cl; 6 College of Horticulture, Sichuan Agricultural University, Chengdu 611130, China; bsun@sicau.edu.cn

**Keywords:** *Lactuca sativa*, polyphenol concentration, light supplementation, light enrichment

## Abstract

The growth and development of green lettuce plants can be modulated by the prevailing light conditions around them. The aim of this study was to evaluate the effect of ambient light enrichment with different LED light spectra on agronomic characteristics, polyphenol concentration and relative gene expression of enzymes associated with polyphenol formation in ‘Levistro’ lettuce grown hydroponically in a Nutrient Film Technique (NFT) system for 28 days in a greenhouse. The spectra (blue:green:red:far-red) and red:blue (R:B) ratios obtained by enriching ambient light with Blue (B), White (W), Blue-Red (BR) and Red (R) LED light were B: 47:22:21:10, 0.5:1; W: 30:38:23:9, 0.8:1; BR: 33:15:44:8, 1.3:1 and R: 16:16:60:8, 3.8:1, respectively, and photosynthetically active radiation (PAR) under the different treatments, measured at midday, ranged from 328 to 336 µmoles m^−2^ s^−1^. The resulting daily light integral (DLI) was between 9.1 and 9.6 mol m^−2^ day^−1^. The photoperiod for all enrichment treatments was 12 h of light. The control was ambient greenhouse light (25:30:30:15; R:B = 1.2:1; PAR = 702 µmoles m^−2^ s^−1^; DLI = 16.9 mol m^−2^ day^−1^; photoperiod = 14.2 h of light). Fresh weight (FW) and dried weight percentage (DWP) were similar among the enrichment treatments and the control. The leaf number increased significantly under BR and R compared to B lights. The relative index of chlorophyll concentration (RIC) increased as plants grew and was similar among the enrichment treatments and the control. On the other hand, the concentration of chlorogenic acid and chicoric acid increased under BR and B lights, which was consistent with the higher relative expression of the *coumarate 3-hydroxylase* enzyme gene. In view of the results, it is inferred that half of the PAR or DLI is sufficient to achieve normal growth and development of ‘Levistro’ lettuce plants, suggesting a more efficient use of light energy under the light enrichment treatments. On the other hand, the blue and combined blue-red lights promoted the accumulation of phenolic compounds in the leaves of ‘Levistro’ lettuce plants.

## 1. Introduction

Advances in light technology have made it possible to commercially grow and improve vegetable production in greenhouses and vertical farms. Specifically, light spectrum variation can be used as a control of morphogenesis [1,2,3], photosynthetic response [3,4] and secondary metabolite production [2,5,6]. Blue and red lights have been considered the most relevant for plant growth and development. Both types of light are the main sources of energy for photosynthetic assimilation of carbon dioxide [3,7]. They influence the growth and morphology of different vegetable species and cultivars [3,8,9], acting alone with each other or in combination with white light [3]. Likewise, blue, red and white lights can be a potent genetic modulator, significantly affecting the production of secondary metabolites, especially those with strong antioxidant activity, such as phenolics and carotenoids [10].

Under greenhouse conditions, LED lighting has been used to supplement the insufficient light that occurs in intensive greenhouse production [11,12]. Likewise, LED lights, due to their characteristics, have allowed for altering the surrounding spectrum where plants are grown by promoting or maintaining the agronomic and physiological characteristics of several vegetable species [11,13,14,15,16,17]. For example, the FW, DWP and SPAD index of tomato plants increased significantly when the ambient greenhouse light was enriched with a red + blue + far-red light, while the leaf area ratio (cm^2^ g FW^−1^) was higher in the treatment where the light was blue plus red compared to plants that did not have ambient light enrichment [11]. In bell pepper plants, there was an increase in the leaf area index but no significant difference in leaf number and SPAD index with the addition of cool-white top-lighting after 56 days of enrichment light application [13]. On the other hand, the leaves of two pepper cultivars were thicker and had larger palisade parenchyma cells under LED enrichment lighting (12.5% blue light and 87.5% red light; R:B = 7:1) compared to leaves grown under high-pressure sodium (HPS) lamps [17]. In lettuce, enrichment of greenhouse ambient light with HPS and LED lamps for 5 days before harvest, regardless of the spectrum, improved the relative chlorophyll content and red color of the four lettuce varieties studied [16]. Other lettuce studies observed no changes in FW or the dried weight of different lettuce cultivars grown in winter under ambient light enrichment with LED (blue or red light) or HPS lamps [14,15].

The antioxidant compounds can help plants act against biotic and abiotic stresses and can be appreciated by consumers for their positive effects on health [18]. Antioxidant compounds such as polyphenols are considered essential functional foods in our diet [19] and exhibit beneficial properties for human health [20]. For instance, dietary polyphenols help improve lipid profiles, blood pressure, insulin resistance, systemic inflammation and cardiovascular health [19]. Particularly, lettuce possesses different polyphenols such as caffeic acid derivatives, quercetin and kaempferol glycosides [20,21] and can be an interesting and inexpensive plant source of antioxidant phenolic extracts for functionalizing foods [22]. The quantity and quality of antioxidant compounds are genetically driven; nonetheless, light is one of the factors that strongly influence their synthesis and accumulation in plant tissues [23]. In general, various bioactive compounds respond differently to light treatment and their biosynthesis is expected to be promoted in a species/cultivar-specific manner [18]. Other factors, such as the amount of light, growing season and metabolic factors may also impact their accumulation [5,6,24,25]. Particularly, the enrichment of ambient light with red light (R:B = 7.5:1.0) diminished the total phenol concentration and antioxidant capacity of green ‘Lavinia’ lettuce in the three growing seasons [24], whereas in green ‘Little gem’ lettuce, the most pronounced positive effect on total phenolic compounds was given by supplemental illumination with blue (400 nm) and blue + green (455 + 530 nm) LEDs [25]. Specific wavelengths can influence the concentration of particular polyphenols in vegetables. For example, in lettuce, blue light stimulated the concentration of chlorogenic acid [26,27,28], although so did blue-red light [28] and red light with an even greater effect than blue light [27]. Additionally, blue LED light added to the HPS lamp in the greenhouse promoted significant differences in quercetin glucuronide and quercetin malonyl glucoside concentration in red ‘Lollo Rosso’ but not in green ‘Batavia’ lettuce [15]. On the other hand, blue + red and white + red + blue promoted higher quercetin concentration in lettuce plants compared to red or white monochromatic light under the same PAR, whereas red lettuce significantly increased quercetin concentration only under red-blue light [29].

Different studies have observed that the accumulation of several polyphenols coincides with the expression pattern of genes associated with their synthesis in lettuce plants under different light spectra [29,30,31,32], suggesting that these genes play important roles in phenylpropanoid and flavonoid biosynthesis. Namely, red lettuce plants grown under mixed light (red + blue + white) showed high expression levels of *cinnamate 4-hydroxylase* (*C4H)*, *flavanone 3-hydroxylase* (*F3H*) and *dihydroflavonol 4-reductase* (*DFR*) genes. Further, plants from the same treatment possessed higher contents of gallic acid, chlorogenic acid and quercetin than plants exposed to single light (red or blue light) [29]. Similarly, ‘Rebelina’ lettuce plants grown for 14 days under red and blue LED light (R:B = 3:1) increased concentrations of caftaric and chicoric acids, isoquercetin and luteolin, in agreement with increased expression of some genes related to the formation of phenolic compounds (*coumarate 3-hydroxylase* (*C3H*) and *DFR*) [30]. On the other hand, chlorogenic acid is enhanced in ‘Green wave’ lettuce under continuous blue compared to red light. In addition, the expression of *phenylalanine ammonia-lyase* (*PAL*), *C4H*, *chalcone synthase* (*CHS*), *chalcone isomerase* (*CHI*(*2*)), *F3H* and *flavonol synthase* (*FLS*) genes was higher under blue than red light [32]. Thereby, the light spectrum can influence the expression patterns of different enzymatic genes associated with polyphenol formation.

There are limited studies to identify the effects of modifying the ambient light spectrum by enriching it with blue, white, blue-red and red light on the growth and secondary metabolism of lettuce plants grown in greenhouses during summer. Particularly, further studies on the effects of spectral quality and light intensity on the polyphenol formation pathway are needed to understand the modulatory role of LED light supplementation on the expression of genes favoring the production of antioxidant molecules, such as polyphenols. In addition, the results of the literature allow us to deduce that the effect of the variation of the light spectrum affects crops differentially depending on the species, cultivar and growing season. Therefore, it is necessary to carry out detailed studies to evaluate the response of a particular crop under different light conditions. Thus, the purpose of this research was to determine the effect of ambient light enrichment with different LED light spectra on agronomic characteristics, polyphenol concentration and relative expression of genes associated with polyphenol formation in green ‘Levistro’ lettuce plants grown hydroponically under greenhouse conditions.

## 2. Results

### 2.1. Agronomic Characteristics

#### 2.1.1. Fresh Weight (FW) and Dried Weight Percent (DWP)

The FW and DWP were similar between treatments and the control. In particular, FW under the different treatments ranged between 35.6 and 45.2 g (Table 1). Meanwhile, DWP ranged between 9.0 and 9.8% (Table 1).

#### 2.1.2. Leaf Number

The leaf number showed significant differences among treatments. Specifically, leaf number was significantly higher under BR (33:15:44:8; 1.3:1) and R (16:16:60:8; 3.8:1) compared to B (47:22:21:10; 0.5:1) by 13.5 and 11.6%, respectively (Table 1).

#### 2.1.3. Relative Index of Chlorophyll Concentration (RIC)

**Figure 1:**
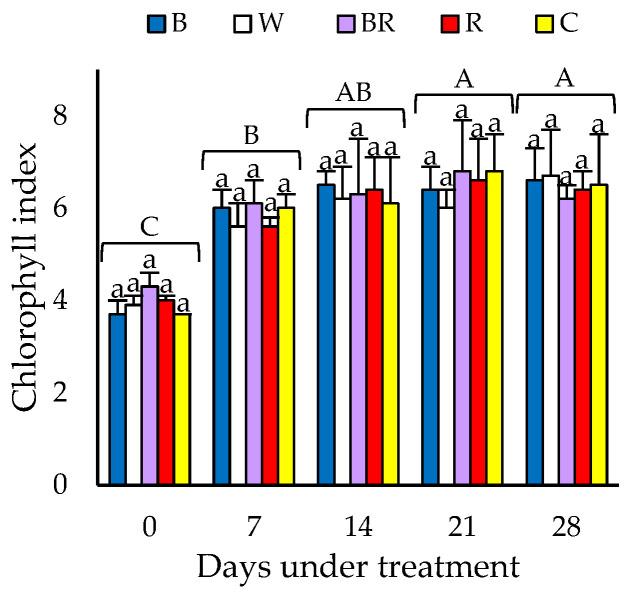
Relative index of chlorophyll concentration of ‘Levistro’ lettuce plants grown hydroponically under ambient light enriched with different LED light spectra. B (blue; 47:22:21:10; 0.5:1), W (white; 30:38:23:9; 0.8:1), BR (blue-red; 33:15:44:8; 1.3:1), R (red; 16:16:60:8; 3.8:1) and C (control; ambient light; 25:30:31:14; 1.2:1). Different lowercase letters indicate significant differences among light spectra and uppercase letters indicate significant differences between evaluation days by Tukey’s test (*p* ≤ 0.05). Mean (*n* = 3) ± SE.

The RIC showed no significant differences among the different light spectra for each day of evaluation. However, significant differences were observed between evaluation days (Figure 1). In detail, RIC increased significantly on days 21 and 28 in comparison with days 7 and 0 by 12.1 and 66.7%, respectively (Figure 1).

### 2.2. Phenolic Profile and Gene Relative Expression

#### 2.2.1. Phenolic Profile

Among the identified phenolic compounds, four belong to the phenolic acid group and three to the flavonoid group. The phenolic acids identified were caftaric acid, chlorogenic acid, caffeoylmalic acid and chicoric acid. Among them, the ones that showed a higher concentration were chlorogenic and chicoric acids (Table 2). Specifically, the concentration of caftaric acid increased significantly under R (16:16:60:8; 3.8:1) with respect to the other light treatments and control, whereas the concentration of chlorogenic acid was significantly higher under BR (33:15:44:8; 1.3:1) and B (47:22:21:10; 0.5:1) compared to the control (25:30:31:14; 1.2:1), W (30:38:23:9; 0.8:1) and R (16:16:60:8; 3.8:1) by 35.6, 29.8 and 19.6%, respectively, for both acids. Similar results were observed for chicoric acid. In this case, the concentration of chicoric acid was significantly elevated under BR (33:15:44:8; 1.3:1) compared to R (16:16:60:8; 3.8:1), control (25:30:31:14; 1.2:1) and W (30:38:23:9; 0.8:1) by 18.9, 12.5 and 10.5%, respectively. It must be considered that all enrichment light treatments had about half of the intensity (330 to 340 μmoles m^−2^ s^−1^) than the control (702 μmoles m^−2^ s^−1^). Although the concentration of chicoric acid under B (47:22:21:10; 0.5:1) was higher than the control (25:30:31:14; 1.2:1), non-significant differences were found (Table 2). Finally, caffeoyl malic acid concentration showed non-significant differences among the treatments (Table 2).

On the other hand, the flavonoids identified were glycosylated quercetin and luteolin. Particularly, the concentration of quercetin-3-O-glucoside increased under control (25:30:31:14; 1.2:1) versus R (16:16:60:8; 3.8:1), BR (33:15:44:8; 1.3:1) and W (30:38:23:9; 0.8:1) by 100, 66.7 and 66.7%, respectively (Table 2). On the other hand, the concentration of quercetin-3-O-glucuronide was higher under the control (25:30:31:14; 1.2:1) relative to R (16:16:60:8; 3.8:1) by 35.7% (Table 2). Similarly, luteolin-7-O-glucoside concentration was higher under the control (25:30:31:14; 1.2:1) than in R (16:16:60:8; 3.8:1), B (47:22:21:10; 0.5:1), BR (33:15:44:8; 1.3:1) and W (30:38:23:9; 0.8:1) by 150, 150, 66.7 and 42.9%, respectively (Table 2).

#### 2.2.2. Gene Relative Expression

**Figure 2:**
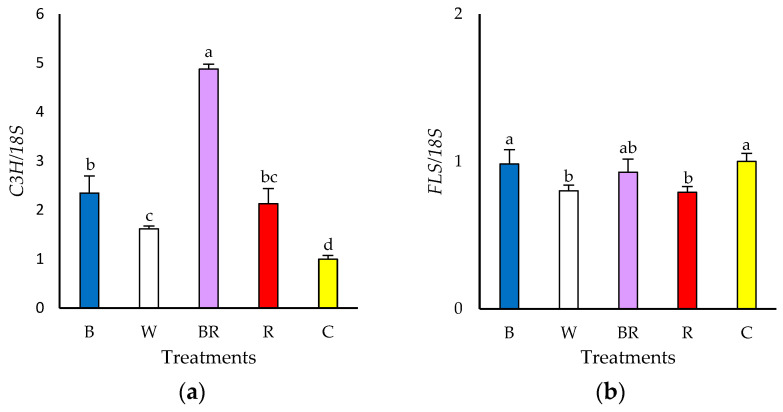
Gene relative expression of ‘Levistro’ lettuce plants grown hydroponically under ambient light enriched with different LED light spectra. (**a**) *Coumarate 3-hydroxylase* (*C3H*) gene relative expression; (**b**) *flavonol synthase* (*FLS*) gene relative expression. *18S* reference gene. B (blue; 47:22:21:10; 0.5:1), W (white; 30:38:23:9; 0.8:1), BR (blue-red; 33:15:44:8; 1.3:1), R (red; 16:16:60:8; 3.8:1) and C (control; ambient light; 25:30:31:14; 1.2:1). Different letters indicate significant differences using Tukey’s test (*p* ≤ 0.05). Mean (*n* = 3) ± SE.

Statistically, all spectra that enriched ambient light showed a significantly higher level of relative expression of the *coumarate 3-hydroxylase* (*C3H*) gene (Figure 2a). Nevertheless, when considering the biological threshold (>2-fold) only BR (33:15:44:8; 1.3:1) (4.9-fold) significantly increased the expression of this gene compared to the control (25:30:31:14; 1.2:1) (1-fold) (Figure 2a). On the other hand, the expression level of *flavonol synthase* (*FLS*) gene was similar among treatments considering the biological threshold (> 2-fold) (Figure 2b). However, statistical analysis showed that both W (30:38:23:9; 0.8:1) (0.8-fold) and R (16:16:60:8; 3.8:1) (0.8-fold) decreased the expression level of this gene compared to the control (25:30:31:14; 1.2:1) (1-fold) (Figure 2b).

## 3. Discussion

### 3.1. Agronomic Characteristics

#### 3.1.1. Fresh Weight (FW), Dried Weight Percentage (DWP) and Leaf Number

The different light enrichment treatments did not cause significant changes in the FW and DWP of lettuce plants compared to the control, which had a higher PAR and DLI of almost twice that of the enrichment treatments (Table 1). Similar results have been observed by other research groups. For example, Ouzounis et al. [15] did not observe a variation in FW and DWP of ‘Batavia’ and ‘Lollo Rossa’ lettuce when greenhouse ambient light was supplemented with blue LED light at low intensity or DLI (45–80 µmoles m^−2^ s^−1^; 0.3–1.8 mol m^−2^ day^−1^). In ‘Boston’ lettuce, no significant differences in FW and dried weight percentage were found when ambient greenhouse light was compared to ambient light with enrichment, using high-pressure sodium lamps and LED lamps that yielded an average total radiation during cultivation of 1100 and 550 µmoles m^−2^ s^−1^ (71.3 and 35.8 mol m^−2^ day^−1^), respectively [14]. When light enrichment treatments were compared under the same PAR at 17 mol m^−2^ day^−1^ (~250 µmoles m^−2^ s^−1^), Hernández et al. [33] noted that light supplementation treatment with LED (20% blue and 80% red) or HPS did not significantly affect the FW of 12 green and red lettuce cultivars. In other species, such as basil, FW and DWP were also not compromised under the different greenhouse light enrichment treatments (20%blue–80%red; UVA–20%blue–80%red; 60%blue–40%red and 20%green–80%red) at the same intensity or DLI (175 µmoles m^−2^ s^−1^; 12.6 mol m^−2^ day^−1^) [34]. According to Hernández et al. [33], the effect of the enriched greenhouse ambient light with HPS or LED seems to be overshadowed by the same background ambient light. This is likely due to a high influx of ambient light, particularly on clear days, combined with the appropriate light transmission through the greenhouse observed during this experiment.

On the other hand, the results of this work would indicate that plants grown under ambient light enriched with different spectra, which had approximately half the PAR or DLI (300 to 336 μmoles m^−2^ s^−1^; 9.1 to 9.6 mol m^−2^ day^−1^) of ambient light (702 μmoles m^−2^ s^−1^; 16.9 mol m^−2^ day^−1^), presented normal growth and similar characteristics to those grown under ambient light. Therefore, according to these results, a PAR of 330 μmoles m^−2^ s^−1^ or DLI of ~9.3 mol m^−2^ day^−1^, independent of the spectrum that enriched the ambient light, was adequate for the cultivation of ‘Levistro’ lettuce. Different studies have indicated that CO_2_ assimilation of lettuce has a linear relationship with PAR up to 400–500 µmoles m^−2^ s^−1^ [35,36,37]; thus, weight gain in ‘Levistro’ lettuce would not be limited under the lower radiation observed in the light enrichment treatments. The above could translate into a more efficient use of light energy under the greenhouse light enrichment treatments. Information provided by Runkle [38] suggested a minimum DLI of 12–14 mol m^−2^ day^−1^ for greenhouse lettuce production. Studies with green lettuce cultivars (‘Hongyeom Jeockchukmyeon’ and ‘Rebelina’) found that PARs of 250 to 290 μmoles m^−2^ s^−1^, resulting in a DLI between 14.4 and 18.8 mol m^−2^ day^−1^, respectively, would be enough to guarantee an optimal yield [39,40], whereas Gavhane et al. (2023) [41] indicated that the optimum DLI for iceberg lettuce grown in an indoor vertical hydroponic system was 11.5 mol m^−2^ day^−1^. These results agree with Kelly et al. (2020) [42], who mentioned that the specific combination of PPFD and photoperiod, variables with which DLI is calculated, can have different effects on plant growth. The results of our study showed that a DLI between 9.1 and 9.6 mol m^−2^ day^−1^, independent of the spectrum, would allow for achieving adequate growth and development in lettuce plants, although it would depend on the cultivar.

On the other hand, ‘Levistro’ lettuce plants grown under BR and R showed a significantly higher number of leaves than plants grown under B. However, this did not translate into a significant increase in FW and DWP. According to Ouzounis et al. [15], green and red lettuce plants grown under ambient light enriched with HPS plus blue light (45–80 µmoles m^−2^ s^−1^; 0.3–1.8 mol m^−2^ day^−1^) were more compact than those plants without blue light addition. Furthermore, blue light can promote leaf thickening [34]. In other green and red lettuce cultivars (Greenstar, Locarno and Rouxai), ambient light enrichment with HPS, which is characterized by a higher red component, promoted greater plant height and diameter than LED enrichment with 20% blue and 80% red light under the same PAR (180 μmoles m^−2^ s^−1^) or DLI (17 mol m^−2^ day^−1^) [33]. Therefore, the results observed in this study suggested a modification in leaf morphology, compensating for the weight of the plants under the different treatments that enriched ambient light.

#### 3.1.2. Relative Index of Chlorophyll Concentration (RIC)

Under the different light enrichment treatments, the RIC was similar to the control despite the differences in PAR or DLI, but as the days elapsed, the RIC increased significantly (Figure 1). Specifically, the relative index of chlorophyll concentration at the beginning (day 0) was significantly lower than the rest of the evaluations. Coincidentally, in greenhouse-grown green butterhead lettuce ‘Lores’, chlorophyll concentration was lower in young compared to mature leaves [43]. In other plant species, lower chlorophyll concentration was also found in younger compared to older leaves, i.e., those that can be called ‘photosynthetically mature’ [44,45,46]. According to Šesták [46], chlorophyll concentration depends on leaf age, as leaf chlorophyll concentration changes proportionally with the variation of leaf structure to growth and development [44]. Younger leaf zones may show a light green or yellowish-green coloration, while mature zones show greener colors [47]. In addition, leaves or areas within leaves that are not yet photosynthetically mature may have little mesophyll tissue, exhibiting a fainter or distinct green color [47,48]. Conversely, mature leaves evidenced more developed and robust mesophyll, exhibiting a green color [49,50]. Therefore, it is possible that the lower RIC in young leaves of ‘Levistro’ lettuce could be due to an incipient development of the internal structure of the photosynthetic organ.

#### 3.1.3. Phenolic Profile and Relative Gene Expression

Among the different phenolic compounds found in nature, Santos et al. [51] indicate that the main phenolic compounds identified in green lettuce leaves correspond to hydroxycinnamic acids. More specifically, Llorach et al. [22] indicated that caffeic acid derivatives were the main phenolic compounds in green lettuce varieties, agreeing with Romani et al. [21], who further indicated that flavonols are another main class of polyphenols in lettuce leaves. For Materska et al. [20], quercetin and caffeic acid derivatives are the main phenolic compounds in lettuce. In our study, the phenolic acids identified were caftaric acid, caffeoylmalic acid, chlorogenic acid and chicoric acid. Among them, the highest concentrations were chlorogenic acid and chicoric acid, which coincided with the results obtained by Materska et al. [20], Romani et al. [21] and Santos et al. [51]. Among the flavonoids, two glycosylated quercetins (quercetin-3-O-glucoside and quercetin-3-O-glucuronide) and luteolin-7-O-glucoside were identified.

Light spectrum is a relevant factor in the formation of secondary metabolites [6,47,48]. Our results showed that chlorogenic acid concentration increased significantly under BR (33:15:44:8; 1.3:1) and B (47:22:21:10; 0.5:1) compared to the control (25:30:31:14; 1.2:1), which possessed a substantially higher PAR or DLI (Table 2). Previous research noted an increase in chlorogenic acid concentration in ‘Green Wave’ lettuce exposed for one week after transplanting to fluorescent or continuous blue LED light compared to continuous red LED light treatment at the same PAR (200 µmoles m^−2^ s^−1^) or DLI (8.6 mol m^−2^ day^−1^) [26]. Similarly, chlorogenic acid concentrations in lettuce seedlings 17 days after sowing were significantly higher under blue LEDs and blue-red light compared to red light and fluorescent light with a PAR of 100 µmoles m^−2^ s^−1^ or DLI of 5 mol m^−2^ day^−1^. However, when exposure was prolonged up to 45 days after sowing, the significant differences disappeared [28]. For their part, Yoshida et al. [27] observed that nighttime supplemental light for 14 h with red and blue LEDs (10–50 µmoles m^−2^ s^−1^; 0.5–2.5 mol m^−2^ day^−1^), three weeks after transplanting, increased chlorogenic acid concentration in ‘Greenwave’ green lettuce compared to those without nighttime supplemental lighting, although the effect of red light was significantly greater than blue light. In ‘Little Gem’ green lettuce plants, supplemental illumination with blue-green (455 + 530 nm) LEDs (50 µmoles m^−2^ s^−1^; 4 h light; 0.7 mol m^−2^ day^−1^) over HPS base light (90 µmoles m^−2^ s^−1^; 1.3 mol m^−2^ day^−1^) in the greenhouse applied during the day in autumn and at night in spring caused a significant increase in chlorogenic acid concentration [25]. In contrast, Taulavuori et al. [52] noted no significant difference in chlorogenic acid concentration of red lettuce ‘Lollo Rossa’ grown in a greenhouse for 48 days under ambient light enriched with HPS or HPS plus blue LED light, both at 300 μmoles m^−2^ s^−1^ for 16 h light (17.3 mol m^−2^ day^−1^). Although the effect of light spectrum on chlorogenic acid concentration may vary according to lettuce cultivar, the results obtained in this work suggest that there is a complementary effect of the blue and red component in inducing chlorogenic acid accumulation in green ‘Levistro’ lettuce plants when the ambient greenhouse light was enriched with BR (BR; 33:15:44:8; 1.3:1). Furthermore, the higher PAR or DLI observed in the control showed a lower chlorogenic acid concentration versus the blue-red light enrichment, indicating that light intensity would not exert a preponderant role on chlorogenic acid concentration. This result would agree with those obtained by Becker et al. [53], who noted that PAR reduction from 410 to 225 μmoles m^−2^ s^−1^ did not influence phenolic acid concentrations in red oak leaf lettuce ‘Eventai’.

On the other hand, under the different light enrichment treatments, glycosylated quercetin was the main flavonol found in ‘Levistro’ lettuce leaves. When spectra enriching ambient light were compared, all showed a lower concentration of total quercetin (sum of identified glycosylated quercetins) with respect to the control. However, only red light enrichment (R; 16:16:60:8; 3.8:1) significantly minimized the concentration of total quercetin (Table 2). In green leaf ‘Two Star’ lettuce, the addition of red light to white fluorescent light (270 μmol m^−2^ s^−1^; 12 h photoperiod or 11.7 mol m^−2^ day^−1^) had a negative effect on the concentration of kaempferol and rutin, compounds belonging to the same group as quercetin, but in red leaf ‘New Red Fire’ lettuce, it exerted an opposite effect when compared to white, white + blue and white + infrared lights [54]. Conversely, red light (90 μmol m^−2^ s^−1^; 16 h light or 5.2 mol m^−2^ day^−1^) raised quercetin concentration in kohlrabi sprouts versus white, blue and blue-red light [55]. In onion, red light together with blue and UV-A light positively promoted quercetin concentration, although not as profoundly as that of white light [56]. Therefore, the effect of red light on flavonoids seems to be cultivar and plant-species-dependent.

The observed changes in polyphenols may be due to the impact of wavelength on genes associated with polyphenol-promoting enzymes [57,58]. Thus, in this investigation, a significant increase (considering biological threshold and statistical analysis) in the relative gene expression *coumarate 3-hydroxylase* (*C3H*) enzyme gene under BR (33:15:44:8; 1.3:1) was observed, as well as a significant increase (considering only statistical analysis) under B (47:22:21:10; 0.5:1). This fact confirms the statement of Pu et al. [59] that the enzyme coumarate 3-hydroxylase (C3H) is a precursor to the formation of chlorogenic acid.

According to the literature, both red and blue light promote the expression of polyphenol pathway genes. For example, the putative *C3H* gene, which codes for coumarate 3′-hydroxylase, was expressed to a greater extent under blue-red LED light compared to fluorescent light (215 μmoles m^−2^ s^−1^; 16 h light or 12.4 mol m^−2^ day^−1^) in green lettuce ‘Rebelina’ plants after 14 days of treatment [30]. Another research reported that blue light (200 μmoles m^−2^ s^−1^; 24 h light or 17.3 mol m^−2^ day^−1^) increased the expression levels of a *C3H* in ‘Green wave’ lettuce after two days of exposure to light treatments, as well as other enzyme genes associated with polyphenol formation, including *phenylalanine ammonia-lyase* (*PAL*), *chalcone synthase* (*CHS*) and *flavonol synthase* (*FLS*) [32]. Similarly, blue LED light increased *PAL* gene expression in red ‘Sunmang’ lettuce at 9 days after transplanting compared to red, green, white and fluorescent lamp + HPS lamp. Nonetheless, when the evaluation was performed 23 days after transplanting, the effect of the light spectrum changed and red and white light exacerbated the expression of the same gene [60]. In another study, red lettuce plants grown under blue + red + white light showed increased expression of *C4H*, *F3H* and *DFR* genes, which coincided with elevated concentrations of gallic acid, chlorogenic acid and quercetin under the same light treatment [29]. Studies in *Arabidopsis thaliana* indicated that phytochrome (PHY) A or B and cryptochrome (CRY) 2 would be the primary photoreceptors involved in light-dependent polyphenol accumulation [61]. The phytochrome molecule is activated by red light [62,63] while cryptochrome receives blue light [64]. Then, both blue light and red light by themselves exert an impact on gene expression and, presumably, blue light and red light could interact in a way that enhances the effect of light on the gene expression of enzymes of the phenolic compound formation pathway through these photoreceptors.

## 4. Materials and Methods

### 4.1. Plant Material and Growth Conditions

The experiment consisted of growing green ‘Levistro’ lettuce plants (Rijk Zwaan, De Lier, The Netherlands) under ambient greenhouse light enriched with different LED light spectra for 28 days post-transplant in summer (growing period between 23 December 2018 to 20 January 2019). The ‘Levistro’ cultivar belongs to the Lollo bionda lettuce type and is characterized by loose leaves that can be harvested individually. Seeds were sown in plastic trays with 196 alveoli containing a mixture of peat (KEKKILÄ professional DSMO W, Vantaa, Finland) and A6 expanded perlite (Harbolite, Santiago, Chile) in a 1:1 (*v*:*v*) ratio. After the seedlings reached 5 to 6 cm root length and three to four true leaves (30 days after sowing), they were transplanted into each hydroponic system. During transplanting, each seedling was placed in a 3 × 3 cm low-density sponge cube (polyfoam).

The experiment was carried out on three independent NFT hydroponic systems. Each system consisted of eight troughs of 0.15 × 7.00 × 0.07 m (width × length × height). Each individual hydroponic system formed a replicate. Forty-six plants per m^2^ were placed in each hydroponic system. Thus, a group of forty plants was transplanted for each light enrichment treatment and replicate. Harvesting was carried out 28 days post-transplanting. Three plants per experimental unit were harvested for the different evaluations.

The nutrient solution used was the one proposed by Lara et al. [65], which was kept in constant recirculation, reaching an average oxygen concentration of 7.5 ± 0.5 mg L^−1^ (Oxyguard Handy Polaris, Farum, Denmark) during the culture period. The average pH was 5.8 ± 0.1 and was measured with a potentiometer (Hi99301, Hanna Instruments, Woonsocket, RI, USA). During the culture period, the pH adjustments were made with an acid solution (1.2% phosphoric acid + 3.8% nitric acid + 95% water). The average electrical conductivity reached 2.2 ± 0.1 mS cm^−1^ and was evaluated with a conductivity meter (Hi99301, Hanna Instruments, USA). The different evaluations of the nutrient solution were performed every other day between 15:00 and 16:00 of the day.

The hydroponic systems were located inside an 8.0 × 33.0 × 5.8 m (width × length × zenithal height) chapel-type greenhouse with a 200 μm-thick polyethylene cover with more than 90% overall light transmission (Proamco, Colina, Santiago, Chile). In addition, the greenhouse belonging to the Center for Post-harvest Studies (CEPOC) of the Faculty of Agronomic Sciences at the University of Chile (latitude: −33.57, longitude: −70.63), had a wet-wall cooling system set at a temperature of 25 °C. The mean temperature and relative humidity during the growing period were 23.1 ± 8.4 °C and 61.5 ± 19.4%, respectively. Both variables were recorded hourly during the study period with RC-51H data loggers (Elitech, London, UK).

### 4.2. Treatments

**Figure 3:**
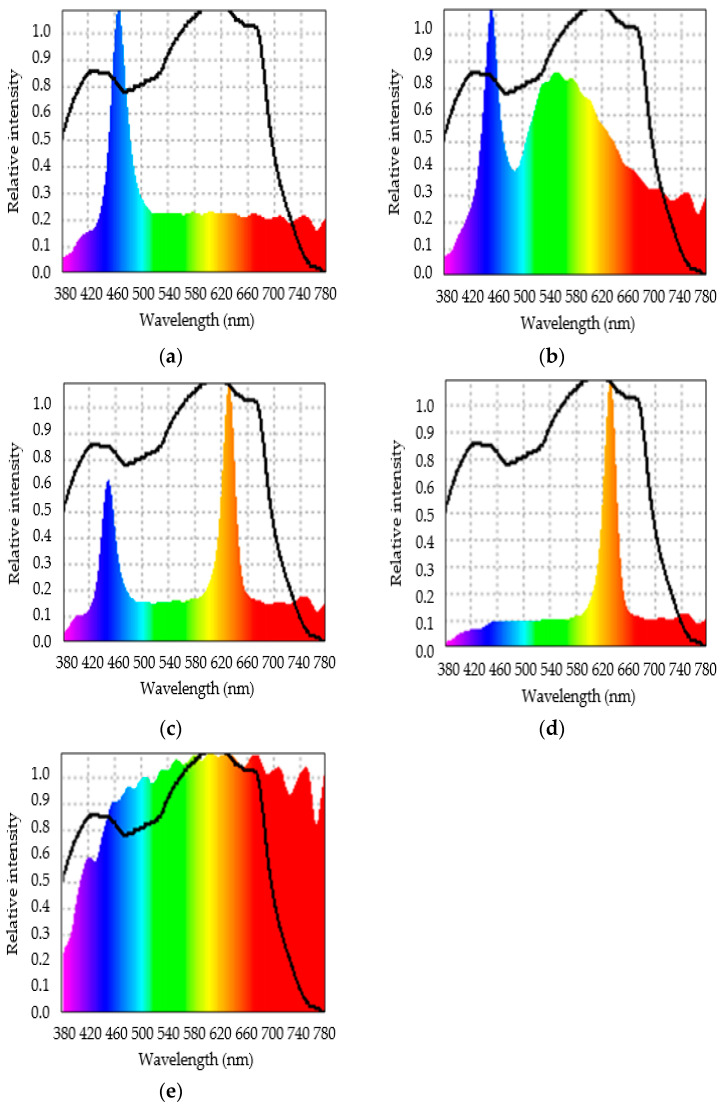
Ambient light enriched with different LED light spectra under which ‘Levistro’ lettuce plants were grown for 28 days: (**a**) ambient light enriched with blue LED light (47:22:21:10; 0.5:1); (**b**) ambient light enriched with white LED light (30:38:23:9; 0.8:1); (**c**) ambient light enriched with blue-red LED light (33:15:44:8; 1.3:1); (**d**) ambient light enriched with red LED light (16:16:60:8; 3.8:1) and (**e**) the control’s ambient light without enrichment (25:30:31:14; 1.2:1). The colors correspond to the wavelengths of the light spectrum. The continuous line in black corresponds to the reference spectrum chosen (McCree’s action spectrum).

**Figure 4:**
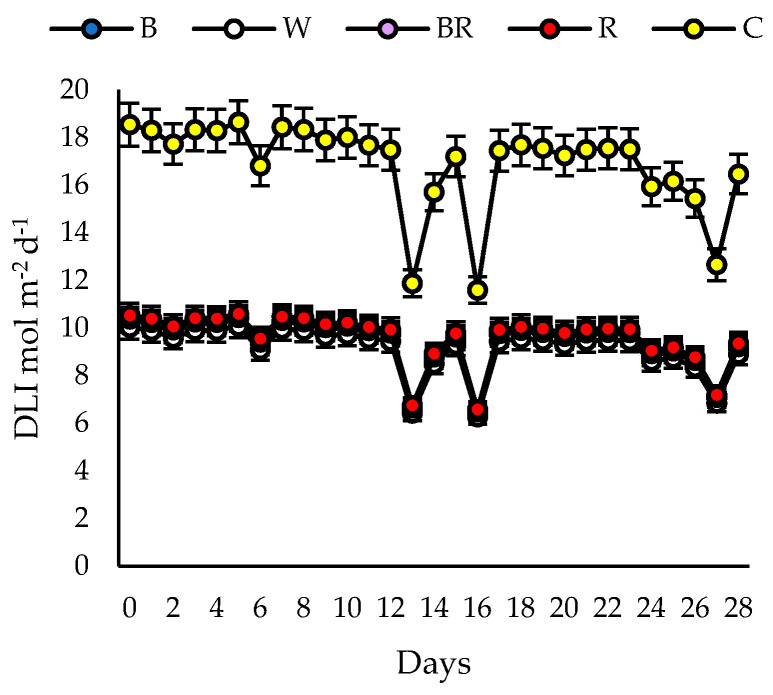
Daily light integral (DLI) under each treatment in which ambient light was enriched with different light spectra for 28 days. B (blue; 47:22:21:10; 0.5:1), W (white; 30:38:23:9; 0.8:1), BR (blue-red; 33:15:44:8; 1.3:1), R (red; 16:16:60:8; 3.8:1) and C (control; ambient light; 25:30:31:14; 1.2:1). Mean (*n* = 3) ± SE.

The light treatments consisted of different LED light spectra that enriched the ambient greenhouse light, and the control was the ambient greenhouse light with no enrichment (Figure 3). The characteristics of the light treatments are detailed in Table 3. The spectra used that enriched the ambient light were blue (B), white (W), blue-red (BR) and red (R). The blue, white and red spectra were obtained from LED lights installed on a 120 × 35 cm wooden panel manufactured by ASYCAR (Santiago, Chile), while the blue-red spectrum was obtained from 36 × 30 cm LED lamps (ASYCAR, Santiago, Chile) mounted on a 120 × 35 cm wooden panel. A spectroradiometer (Asense Tek, Taiwan) associated with the Spectrum Genius Agricultural Lighting Application was used to determine the light spectra in the 380–780 nm range and PAR for each treatment and replicate. The lamps were placed at a height of 30 cm above the plants and the PAR was adjusted using a dimmer to reach between 330 and 340 μmoles of photons m^−2^ s^−1^ at plant level at midday. DLI was calculated under each treatment with data obtained from SSR3D solar radiation sensors (HOBO, Massachusetts, USA) connected to H21-USB data loggers (HOBO, Massachusetts, USA) (Figure 4). Moreover, LED light treatments were spatially separated from each other by a distance of 0.5 m in each hydroponic system. Lamps were turned on for 12 h d^−1^ (8:00 to 20:00 h) for all enrichment treatments and replicates. The photoperiod for the control (greenhouse ambient light) averaged 14.2 h d^−1^ during the growing period.

### 4.3. Evaluations

The evaluations related to agronomic characteristics (FW, DMP and leaf number) were carried out at harvest, while the RIC were evaluated every 7 days, starting at the initial evaluation (day 0) three days post-transplanting until harvest (28 days after ambient light enrichment was applied).

#### 4.3.1. Fresh Weight (FW)

FW was obtained from the aerial part of three independent plants for each replicate and treatment. An analytical balance (RADWAG, AS/100/C/2, Radom, Poland) was used and the weight was recorded in grams (g).

#### 4.3.2. Dried Weight Percentage (DWP)

The aerial part of three independent plants from each replicate and treatment was weighed on an analytical balance (RADWAG, AS/100/C/2, Radom, Poland) and the FW was recorded. The leaves were then dried at 70 °C in an air-circulating oven (LabTech, model LDOS50F, Hwado-eup, Korea) until the dried weight was constant. DWP was determined by the following equation:

DWP = (DW)/FW) × 100(1)

where FW is fresh weight and DW is dried weight.

#### 4.3.3. Leaf Number

The total number of leaves of three independent plants for each repetition and treatment was counted at the time of harvest.

#### 4.3.4. Relative Index of Chlorophyll Concentration (RIC)

RIC was estimated with a CCM-200 plus meter (Opti-Sciences Inc., Hudson, NY, USA) on the third or fourth leaf of three independent plants of each replicate and treatment. Three points of each leaf were evaluated (apex and both sides of the leaf lamina). The evaluations were carried out on days 0, 7, 14, 21 and 28. The results corresponded to the average of three plants for each replicate and treatment.

#### 4.3.5. Phenolic Profile

Extract: The extract was obtained from 100 mg of lyophilized leaf obtained at harvest (28 days after ambient light enrichment). To the extract was added 3 mL of 70% methanol. The mixture was sonicated for 5 min, then centrifuged at 7300× *g* for 10 min at 4 °C (Hermle Brand, model Z326K, Wehingen, Germany). The recovered supernatant was concentrated under vacuum at <35 °C to 50% of its initial volume in a centrifugal concentrator (Centrivap Labconco, Kansas, MO, USA). It was then brought to a 2 mL volumetric capacity with the methanol:water mixture (70:30). Finally, the solution was filtered with 0.45 µm membrane filters (HAWP04700, Millipore, Burlington, MA, USA).

Phenolic Profile: The phenolic compounds present in the filtered extracts of the samples were analyzed using a high-performance liquid chromatograph equipped with a 991 diode array detector (Waters Corp., Milford, MA, USA). Separation was performed on a Waters Nova-Pak C18 (300 × 3.9 mm, 4 mm) reversed-phase column (Millipore, Milford, MA, USA) at room temperature. Two mobile phases were used for elution: A (water:acetic acid (98:2), *v*:*v*)) and B (water:acetonitrile:acetic acid (78:20:2, *v*:*v*:*v*:*v*)). The gradient profile was 0–55 min, 100–20% A; 55–70 min, 20–10% A; 70–90 min, 10–0% A. A flow rate of 0.7 mL min^−1^ together with an injection volume of 10 µL was used. Detection was carried out at 280 nm, while quantification was performed using external standard calibration curves. The results were expressed as mg g DW^−1^.

#### 4.3.6. RNA Isolation and Relative Expression of Genes

Total RNA was isolated from a frozen and powdered sample of the same samples used for phenolic profiling for each replicate and treatment, using TRIzol™ reagent (Invitrogen) according to the manufacturer’s instructions. Total RNA was quantified using an Epoch microplate spectrophotometer (BioTek Instruments, Bad Friedrichshall, Germany). The quality and integrity of total RNA were checked by electrophoresis on a 1.5% agarose gel stained with red gel dye (Biotium, Fremont, CA, USA). Total RNA (1 µg) was reverse transcribed using an ALL-IN-ONE 5X RT MasterMix cDNA synthesis kit (Applied Biological Materials, Richmond, BC, Canada).

Relative quantifications of lettuce transcripts were determined by real-time polymerase chain reaction (RT-qPCR) using three biological replicates with three technical replicates. RT-qPCRs were performed on an Eco™ Real-Time PCR System thermal cycler (Illumina, Inc., San Diego, CA, USA) using the EvaGreen^®^ Dye, 20X in Water kit (Biotium, Fremont, CA, USA) following the manufacturer’s instructions. RT-qPCRs were started with a denaturing temperature at 94 °C for 30 s followed by 40 cycles, using the conditions mentioned in Table 4.

Relative quantifications of the lettuce transcripts were determined by real-time polymerase chain reaction (RT-qPCR) using three biological replicates. The reference primer (18S) and the primer for the gene of interest, *coumarate 3-hydroxylase* (*C3H*), were designed using Primer3web program version 4.1.0 “https://primer3.ut.ee/ (accessed on 20 June 2018), verifying the absence of secondary structures, while the *flavonol synthase* (*FLS*) interest gene was obtained from the literature [23]. In addition, a BLAST was performed with the lettuce genome to confirm that these primers aligned with the genes of interest. Subsequently, PCR efficiency was determined by applying a linear regression analysis to the exponential phase of the amplification curve for each PCR reaction through the LinRegPCR program [66,67]. The mean PCR efficiency for the reactions of each replicate per treatment was normalized by using the 2^−∆∆CT^ method [65]. The list of primers used in this study is detailed in Table 5.

The results were expressed as the ratio between the transcript levels in the samples under the light enrichment treatments versus the samples under ambient light considered as control (fold change). The analysis of the results was based on a biological significance whose minimum threshold of difference in gene expression was 2-fold between treatments, as reported by Ma et al. [57] and Mao et al. [68].

### 4.4. Experimental Design and Statistical Analysis

The experiment was set up in a completely randomized block design with three replicates. Each replicate was an independent NFT hydroponic system where the different light enrichment treatments were randomized. The experimental unit consisted of 40 plants, and an observational unit of 18 plants was used. For each evaluation, three plants were chosen from each replicate. Additionally, a 5 × 5 factorial structure was added for the ICR evaluation, where the first factor corresponded to the light enrichment treatments (B, W, BR, R and the control) and the second level corresponded to the evaluation days (0, 7, 14, 21 and 28). Results were presented as mean values ± standard error (SE). Data were evaluated by analysis of variance (ANOVA) and differences between means were compared using Tukey’s test (*p* ≤ 0.05). Statistical analyses were performed with InfoStat version 2008.

## 5. Conclusions

Varying the light spectrum under greenhouse conditions modified the phenolic profile and gene expression of enzymes associated with polyphenol formation in green ‘Levistro’ lettuce. Enrichment of ambient light with BR (33:15:44:8; 1.3:1) at 336 µmol m^−2^ s^−1^ was the most effective in positively promoting the concentration of phenolic acids, especially chlorogenic acid, through a higher relative expression of the *coumarate 3-hydroxylase* (*C3H*) enzyme gene associated with its formation than in non-enrichment ambient light at 702 µmol m^−2^ s^−1^. On the other hand, the agronomic characteristics of ‘Levistro’ lettuce plants in the greenhouse were not affected by the different light enrichment treatments, indicating that photosynthetically active radiation or DLI equivalent to almost half that observed in ambient light (330 to 336 compared to 702 µmoles m^−2^ s^−1^ or 9.1 to 9.6 compared to 16.9 mol m^−2^ day^−1^, respectively) is sufficient for optimal growth and development of ‘Levistro’ lettuce plants. This makes it possible to assume significant energy savings for lettuce production with a view to cultivation in fully controlled chambers.

## Figures and Tables

**Table 1:** Agronomic characteristics of ‘Levistro’ lettuce plants grown hydroponically under ambient light enriched with different LED light spectra.

Treatments			DLI ^1^	PAR ^2^	Fresh Weight	Dried Weight	Leaf Number
Light	Spectrum	R:B Ratio ^3^	mol m^−2^ day^−1^	µmoles m^−2^ s^−1^	g plant^−1^	%	plant^−1^
Blue (B)	47:22:21:10	0.5:1	9.4 ± 1.1	331 ± 26	35.6 ± 3.3 a ^4^	9.1 ± 0.2 a	14.7 ± 0.6 b
White (W)	30:38:23:9	0.8:1	9.1 ± 1.0	330 ± 25	38.3 ± 5.9 a	9.2 ± 0.4 a	15.3 ± 1.2 ab
Blue-Red (BR)	33:15:44:8	1.3:1	9.5 ± 1.1	336 ± 20	41.4 ± 3.4 a	9.0 ± 0.6 a	16.7 ± 1.0 a
Red (R)	16:16:60:8	3.8:1	9.6 ± 1.1	328 ± 24	40.6 ± 7.1 a	9.8 ± 0.7 a	16.4 ± 0.7 a
Control ^5^	25:30:31:14	1.2:1	16.9 ± 1.9	702 ± 126	40.6 ± 7.0 a	9.1 ± 0.3 a	16.2 ± 0.8 ab

^1^ Daily light integral; ^2^ photosynthetically active radiation measured at midday; ^3^ red:blue ratio; ^4^ different letters in the same column indicate significant differences using Tukey’s test (*p* ≤ 0.05). Mean (*n* = 3) ± SE; ^5^ ambient light.

**Table 2:** Phenolic compound concentration of ‘Levistro’ lettuce plants grown hydroponically under ambient light enriched with different LED light spectra.

Treatments			DLI ^1^	PAR ^2^	Caftaric Acid	Chlorogenic Acid	Caffeoylmalic Acid	Chicoric Acid	Quercetin-3-O-glucoside	Quercetin-3-O-glucuronide	Total Quercetin	Luteolin-7-O-glucoside
Light	Spectrum	R:B Ratio ^3^	mol m^−2^ day^−1^	µmoles m^−2^ s^−1^	mg g^−1^ Dried Weight							
Blue (B)	47:22:21:10	0.5:1	9.4 ± 1.1	331 ± 26	0.3 ± 0.0 c ^4^	6.1 ± 0.6 a	0.5 ± 0.1 a	5.9 ± 0.2 ab	0.8 ± 0.2 ab	1.8 ± 0.3 ab	2.5 ± 0.4 ab	0.4 ± 0.1 c
White (W)	30:38:23:9	0.8:1	9.1 ± 1.0	330 ± 25	0.3 ± 0.0 c	4.7 ± 0.4 b	0.5 ± 0.1 a	5.7 ± 0.3 b	0.6 ± 0.2 bc	1.6 ± 0.3 ab	2.3 ± 0.2 ab	0.7 ± 0.1 b
Blue-Red (BR)	33:15:44:8	1.3:1	9.5 ± 1.1	336 ± 20	0.3 ± 0.0 c	6.1 ± 0.4 a	0.5 ± 0.1 a	6.3 ± 0.6 a	0.6 ± 0.2 bc	1.6 ± 0.3 ab	2.2 ± 0.3 ab	0.6 ± 0.1 b
Red (R)	16:16:60:8	3.8:1	9.6 ± 1.1	328 ± 24	0.4 ± 0.0 a	5.1 ± 0.4 b	0.6 ± 0.0 a	5.3 ± 0.5 b	0.5 ± 0.1 c	1.4 ± 0.4 b	1.9 ± 0.5 b	0.4 ± 0.1 c
Control ^5^	25:30:31:14	1.2:1	16.9 ± 1.9	702 ± 126	0.4 ± 0.0 bc	4.5 ± 0.3 b	0.5 ± 0.0 a	5.6 ± 0.4 b	1.0 ± 0.2 a	1.9 ± 0.2 a	3.0 ± 0.2 a	1.0 ± 0.1 a

^1^ Daily light integral; ^2^ photosynthetically active radiation measured at midday; ^3^ red:blue ratio; ^4^ different letters in the same column indicate significant differences using Tukey’s test (*p* ≤ 0.05). Mean (*n* = 3) ± SE; ^5^ ambient light.

**Table 3:** Detail of the characteristics of the light treatments under which the ‘Levistro’ lettuce plants were grown for 28 days.

Light	Spectrum Blue:Green:Red:Far-Red	Red:Blue Ratio (R:B)	DLI ^1^	PAR ^2^
			mol m^−2^ day^−1^	µmol m^−2^ s^−1^
Blue (B)	47:22:21:10	0.5:1	9.4 ± 1.1	331 ± 26
White (W)	30:38:23:9	0.8:1	9.1 ± 1.0	330 ± 25
Blue-Red (BR)	33:15:44:8	1.3:1	9.5 ± 1.1	336 ± 20
Red (R)	16:16:60:8	3.8:1	9.6 ± 1.1	328 ± 24
Control (ambient light)	25:30:31:14	1.2:1	16.9 ± 1.9	702 ± 126

^1^ Daily light integral (DLI) under each treatment in which ambient light was enriched with different light spectra for 28 days. ^2^ Average photosynthetically active radiation measured at midday during the 28 days that lettuce plants were grown under the different light treatments.

**Table 4:** Conditions for RT-qPCR.

Gene	Annealing		Extension	
	Temperature	Time	Temperature	Time
	°C	s	°C	s
*18S * ^1^	60	30	72	8
*C3H * ^2^	58	30	72	9
*FLS * ^3^	56	30	72	9

^1^ Reference gene; ^2^ *coumarate 3-hydroxylase* enzyme gene; ^3^ *flavonol synthase* enzyme gene.

**Table 5:** Sequence of the primers used for real-time relative quantification.

Gene	5′→3′	3′→5′	Length
			bp
*18S * ^1^	GCC TAC TAT GGT GGT GAC GG	CTA CCT CCC CGT GTC AGG AT	129
*C3H * ^2^	CAA GAA GAG CTC GAC CGT GT	TTG CAT TGG CTT TGT GTG GG	148
*FLS * ^3^	CCA TAC AGA ATA TGT CCT CCA TCA CC	GCT CAA TAT GTC CAT TTG GTC ACC	146

^1^ Reference gene; ^2^ *coumarate 3-hydroxylase* enzyme gene; ^3^ *flavonol synthase* enzyme gene.

## Data Availability

Data are contained within the article.
